# Sleep Bruxism and Hypobaric Hypoxia Exposure: Exploring the Physiological Association

**DOI:** 10.3390/jcm14207176

**Published:** 2025-10-11

**Authors:** Eduardo Pena, Maria Paz Yanez, Francisca Montini

**Affiliations:** 1 High Altitude Medicine Research Center (CEIMA), Arturo Prat University, Iquique 1100000, Chile; 2 Faculty of Health Science, Arturo Prat University, Iquique 1100000, Chile; mariapaz.yanezcarrillo@gmail.com; 3 Health Division of the Chilean Army, Santiago 8340518, Chile; francisca.montini@gmail.com

**Keywords:** high altitude, bruxism, gastroesophageal reflux, hypoxia

## Abstract

Sleep bruxism (SB) is a masticatory muscle activity during sleep which can be categorized as primary, when it remains unclear whether the phenomenon is directly linked to a specific disorder, or if it only coexists, and secondary, when it is proven to be associated with a particular disorder, treatment or lifestyle and bruxism that is part of the signs of a disorder. In this way, SB is associated with various factors, including obstructive sleep apnea and gastroesophageal reflux (GER), where evidence suggests SB has a protective role in airway patency, potentially triggered by microarousals and autonomic instability, especially under hypoxia conditions. Since hypobaric hypoxia exposition—generated by high-altitude exposure—produces a decrease in the partial pressure of oxygen, it triggers alterations in cardiac rhythm and gastric function, which could be associated with physiological alterations mentioned in SB. Therefore, the aim of this review is to determine the effect of hypobaric hypoxia exposure on the physiological and molecular alterations during sleep bruxism. Method: The SANRA-guided narrative review synthesized recent human and animal studies on hypoxia’s physiological and molecular effects in sleep bruxism. In conclusion, SB is associated with GER and autonomic dysregulation, which are present in hypobaric hypoxia conditions, where respiratory disturbances, microarousals, and increased muscle activity are associated with SB. High-altitude exposure triggers oxidative stress, genetics, and sleep alteration, which exacerbate its severity. Moreover, neurophysiological and molecular mechanisms, including TRPV1 and HIF-1α activation, are implicated. Finally, polysomnography remains the gold standard for diagnosis; however, studies at high altitude are needed to confirm this association.

## 1. Introduction

Sleep bruxism (SB) is a masticatory muscle activity that occurs during sleep and is characterized as rhythmic (phasic) or nonrhythmic (tonic), but it is not classified directly as a movement disorder or sleep disorder [1]. Bruxism refers to a group of different jaw-muscle activities during sleep or wakefulness that can result in various motor behaviors, expressed either with or without tooth contact [2]. Current evidence [3] indicates that the overall prevalence of bruxism (both during sleep and wakefulness) is approximately 22%, of which SB constitutes a minor portion (21%) as determined by electromyography or self-reports (with or without clinical examination); however, the prevalence varies when polysomnography is used as a diagnostic method, when the occurrence of sleep bruxism increases to 43%. The reported variations are likely due to differences across studies, which may involve population demographics, diagnostic criteria, or methodological approaches.

Various classification systems have been suggested to describe bruxism, considering the interplay of different contributing factors. For instance, in some cases, bruxism activity may be physiological, whereas in other cases, bruxism may be related to, or a manifestation of, an underlying condition or disorder [2]. In this way, bruxism can be categorized as primary when it remains unclear whether the phenomenon is directly linked to a specific disorder or if it only coexists or secondary when it is demonstrated to be associated with a particular disorder, treatment or lifestyle and when the bruxism is a sign of the disorder [1]. Consequently, in accordance with the clinical effects on orofacial structures, bruxism is considered a risk factor, a protective factor, or an innocuous factor [1] and is also related to the other disorders mentioned above. Since the effects of bruxism can vary from exacerbation of periodontal diseases and destruction of occlusal restorations to contributing as risk factors or aggravating orofacial musculoskeletal symptoms or respiratory pathology, in this context, one study proposed the definition of “patobruxism” for conditions related to orofacial pain, obstructive sleep apnea (OSA), and/or pathological impact on either oral or general health conditions. On the other hand, the term “normo-bruxism” is associated only with normal homeostasis and no signs or symptoms [4]. However, more studies are necessary to determine the principal classification of bruxism.

Currently, studies report that the etiology of SB may include a series of individual factors, such as personality traits, the capacity for adaptability to anxiety and stress, environmental and genetic factors, gene polymorphisms in serotonin and dopamine pathways, biological rhythms, anatomical characteristics, instability in sleep, upper airway patency, and sleep disorders [5,6,7,8]. In recent years, there has been significant interest in exploring the relationship between SB and OSA, a normobaric intermittent hypoxia, because of its possible strong association with biological concomitant alterations [9]. However, there is another kind of hypoxia that is generated by environmental factors, hypobaric hypoxia, which is a condition caused by exposure to high altitudes (2500 m above sea level) and is a product of a decrease in the partial pressure of oxygen (PaO_2_). Diminishing the bioavailability of this vital gas to humans can lead to cardiovascular [10], metabolic [11] and cognitive alterations [12].

Consequently, as mentioned previously, SB is associated with several physiological alterations, i.e., sleep disorders, cardiovascular alterations, changes in cardiac rhythm, genetic factors, anxiety, and stress, which may be exacerbated by hypoxic conditions [13]. With respect to chronic hypobaric hypoxia, this condition has been shown to be associated with various health issues, including sleep disturbances, central nervous system disease, anxiety, and emotional distress [14]; these alterations can be exacerbated by SB, which acts more as a risk factor than as an innocuous or a protective factor, as discussed in the next sections. Additionally, a human study (288 adult volunteers) by Calderón-Jofré et al. [15] of a particular type of hypobaric hypoxia, termed chronic intermittent hypobaric hypoxia (CIHH), which involves alternating days of exposure to high-altitude and high-sea-level conditions, revealed that CIHH can affect sleep quality and melatonin levels, which are crucial for regulating sleep cycles. Moreover, other factors are present in hypobaric hypoxia—such as activation of the sympathetic nervous system, which was corroborated by a human study at 5620 m for 4 weeks [16], and an increased heart rate [10], where upper airway obstruction or hypoxia has been associated with gastroesophageal alterations [16] and where these factors may contribute to or stimulate the SB process. Therefore, the aim of this review is to determine the effects of hypobaric hypoxia exposure on physiological and molecular alterations during sleep bruxism.

## 2. Materials and Methods

This narrative review was conducted following the SANRA guidelines [17] to evaluate the effects of hypobaric and normobaric hypoxia on the physiological and molecular alterations associated with SB. A systematic search was performed in internationally recognized databases (Web of Science, PubMed, Scopus and Google Scholar) and peer-reviewed scientific books using keywords such as sleep bruxism, hypobaric hypoxia, high-altitude exposure, normobaric hypoxia, obstructive sleep apnea, autonomic dysregulation, and molecular mechanisms. The inclusion criteria included studies published within the past five years, animal models with hyperreactivity to hypobaric hypoxia, human and animal studies under hypobaric or normobaric hypoxia conditions, and publications in indexed journals or recognized scientific sources. The exclusion criteria included studies older than ten years, nonindexed journals, and studies lacking original data. Relevant data on study design, population characteristics, exposure conditions, and reported physiological or molecular outcomes were extracted and synthesized narratively, considering heterogeneity in study designs and outcomes. This review integrates evidence on autonomic dysregulation, gastroesophageal reflux, sleep microarousals, and molecular pathways such as TRPV1 and HIF-1α activation, providing a comprehensive overview of SB under hypoxic conditions.

## 3. Sleep Bruxism Mechanism Associated with Gastroesophageal Reflux

Notably, bruxism may also play a physiological role. For instance, jaw movements in SB are related to the ability to secrete stimulated saliva that improves lubrication of the oral cavity and pharynx, which is potentially beneficial during sleep in humans (28.2 ± 8.6 years) [18]. Gastroesophageal reflux (GER) occurs when stomach contents return to the esophagus because of dysfunction of the lower esophageal sphincter (LES). GER is commonly classified into two categories: nonerosive reflux disease (NERD), characterized by reflux-related symptoms in the absence of visible mucosal injury, and erosive reflux disease, defined by the presence of endoscopically confirmed esophageal erosions. Although reflux esophagitis occurs more frequently in men overall, NERD is disproportionately observed in women; additionally, age, habits and pharmacological treatment are important factors to consider, as from the age of 50 and above, tobacco consumption, alcohol intake and pharmacological treatment (aspirin and other nonsteroidal anti-inflammatory agents) increase the risk of developing GER. Epidemiological data indicate that the prevalence of GERD in Western countries ranges from 10–20%, with severe manifestations affecting approximately 6%, whereas in Asian countries, the prevalence is estimated to be approximately 5% [19].

Under physiological conditions, the LES relaxes transiently to allow the passage of food and then contracts to prevent reflux. During sleep, the tone of the LES increases, reducing the likelihood of reflux episodes in patients with SB [20]. Individuals with gastroesophageal reflux may experience inappropriate or sustained relaxations of the LES, decreased esophageal motility, and increased intra-abdominal pressure. During sleep, protective mechanisms such as salivation and swallowing also decrease, which prolongs the exposure and contact time of the esophageal mucosa with acidic contents. As a result, nocturnal reflux tends to be more harmful and more difficult to compensate for patients with GER [21].

Recent studies have suggested that nocturnal GER may act as a trigger for sleep bruxism episodes through a protective reflex mechanism. This physiological sequence has been described as the "triple event model", which includes three consecutive phases: First, during an episode of acid reflux, acid ascends through the esophagus, irritating the esophageal mucosa, which is termed the "initial event"; then a cortical microarousal occurs, in which the irritation elicits a brief cortical activation (detected by electroencephalography), accompanied by changes in autonomic activity, such as increased heart rate; and finally, the orofacial motor response, which is an episode of rhythmic masticatory muscle activity (RMMA), occurs, promoting salivation and swallowing and contributing to the neutralization of the acidic content in the esophagus. This sequence has been observed in studies using polysomnography combined with esophageal pH monitoring, which revealed that episodes of acid reflux precede several bruxism events [22].

Consequently, this phenomenon involves activation of cortical and motor centers that elicit mandibular movements (RMMA), often accompanied by increased swallowing and salivation—responses that may serve to neutralize or clear acidic refluxate. However, the evidence positing a causal link between GERD and SB remains heterogeneous and largely observational, so the proposition that managing GER is a therapeutic strategy for SB should be regarded as provisional. Recent systematic reviews and clinical case series demonstrate associations and plausible pathophysiological mechanisms, yet they also highlight methodological limitations, confounding (e.g., from obstructive sleep apnea), and lack of meta-analytic synthesis or formal grading of evidence necessary to support robust conclusions [20,23,24,25,26].

At the molecular and neurophysiological level, acid exposure in the esophagus appears capable of activating nociceptive receptors (e.g., TRPV1-expressing fibers) and inducing vagal afferent signaling to the nucleus tractus solitarius (NTS); integration of these signals may precipitate microarousals and recruitment of masticatory motor circuits culminating in RMMA. Experimental work has shown upregulation of sensory and neuroinflammatory markers in esophageal mucosa and functional involvement of TRPV1 channels in visceral sensory transmission, providing a biologically plausible substrate for the reflexive and pre-RMMA responses. Nonetheless, most findings are still indirect (animal models, e.g., rat (*Mus musculus*), tissue studies, clinical correlations) and require further validation through longitudinal, interventional, or multimodal human research [27,28,29,30].

The so-called “triple event model”—mucosal irritation → vagal activation → motor response—provides a conceptual framework through which esophageal acid exposure might trigger mandibular movements via glutamatergic and cholinergic neurotransmission, ultimately engaging the trigeminal motor nucleus (MotN) in the brainstem, which is critical for initiating RMMA. Recent polysomnographic studies confirm that RMMA episodes are frequently associated with microarousals and with preceding cortical EEG changes, supporting a reflex–cortical–motor chain; however, the mechanistic evidence linking each step—from acid exposure to specific synaptic activation in the MotN—is still incomplete and fragmented, indicating a need for research integrating neuroanatomical tracing, esophageal sensitivity measurements, and simultaneous VEMG/PSG recordings [31,32,33,34].

Moreover, previous studies have shown the overexpression, at the brainstem level, of a proto-oncogene termed cellular-Fos (c-Fos) after acid reflux, which triggers the activation of visceral–sensory circuits and then SB [35]. In addition, these last two effects involve glutamatergic and GABAergic signaling that interacts with hypothalamic centers to regulate arousal and oral motor activity [35,36]. With respect to acid reflux, there are neuropeptides such as substance P and calcitonin gene-related peptide (CGRP) that can reinforce nociceptive signaling and modulate orofacial motor output through their action in reticular formation and TGV under these acidic conditions [37], which consequently contributes to RMMA and SB.

Notably, mild hypoxia—such as that experienced at high altitudes—can increase sympathetic activation and plasma noradrenaline (NA) levels [38], thereby potentiating oral motor reflexes. A key hypoxia-regulated transcription factor, hypoxia-inducible factor-1α (HIF-1α), is stabilized under hypobaric and normobaric hypoxia conditions and modulates multiple molecular pathways associated with inflammation, including those involving interleukin-6 (IL-6) and nuclear factor-kappa B (NF-κB) and various kinase pathways, such as those involving matrix metalloproteinase-1 (MMP-1), matrix metalloproteinase-3 (MMP-3), lysyl oxidase (LOX), A disintegrin and metalloproteinase with thrombospondin motifs-1 (ADAMTS-1), and angiotensin-converting enzyme (ACE); therefore, HIF-1α is present in several physiological and pathological processes under hypoxic conditions [39]. Furthermore, HIF-1α has been implicated in neuronal plasticity [40] and in alterations in visceral sensitivity, such as gastroesophageal inflammation (NF-κB) and oxidative stress caused by exacerbated levels of reactive oxygen species (ROS) under hypoxic conditions, where both effects have been linked to GER and subsequently SB [19,21,41], which was corroborated in residents living at high altitudes, such as Tibetans, through histopathological, immunohistochemical and ultrastructural analyses of gastric biopsies [39]. Therefore, on the basis of the evidence discussed above, HIF-1α may play a critical role in the development of sleep bruxism (SB) under high-altitude conditions. By modulating inflammatory pathways, including the IL-6 and NF-κB pathway, and influencing oxidative stress (ROS), HIF-1α contributes to alterations in oral motor activity and autonomic regulation, thereby potentially exacerbating SB in individuals in hypobaric hypoxic environments. This highlights the importance of further investigating HIF-1α as a key molecular mediator linked to high-altitude hypoxia, inflammation, and SB pathophysiology; this will be discussed in the next section.

## 4. Hypobaric Hypoxia and Gastroesophageal Reflux

Gastroesophageal alterations associated with GER may be linked to hypoxia. A study by Michaud et al. [42] using an animal model (newborn lambs; *n* = 17) under normobaric hypoxia conditions (10% O_2_ for 3 h) demonstrated that the presence of upper airway obstruction (UAO) for 6 h moderately increased the frequency of GER episodes. Additionally, clinical evidence supports a strong association between oxygen desaturation and GER in patients with primary respiratory symptoms, via simultaneous 24-h multichannel intraluminal impedance (IIM)–pH monitoring and continuous oxygen saturation (SpO_2_) monitoring using pulse oximetry [43].

However, distinguishing between normobaric and hypobaric hypoxia is crucial, as they induce significantly different physiological and molecular responses. Normobaric hypoxia involves a reduced oxygen concentration at sea-level pressure, typically achieved by decreasing the fraction of inspired oxygen (FiO_2_). In contrast, hypobaric hypoxia occurs at reduced atmospheric pressure, simulating high-altitude conditions. These differences lead to distinct ventilatory, cardiovascular, and biochemical adaptations. For instance, compared with normobaric hypoxia, hypobaric hypoxia often results in greater hypoxemia, hypocapnia, and alkalosis because of increased respiratory frequency and lower tidal volume under low-pressure conditions. Additionally, hypobaric hypoxia has been associated with more pronounced oxidative stress and differential gene expression related to hypoxia-inducible factors (HIFs) and mitochondrial function in Sprague-Dawley rats (*n* = 6) under exposure for 6 h at a simulated altitude of 25,000 feet (approximately 7620 m) with barometric pressure reduced to 282 mmHg and exposure for 6 h to an oxygen concentration of 8% (FiO_2_) under normobaric conditions [44] and in a human study (27.6 ± 6.2 years) under normobaric hypoxia with a partial pressure of inspired oxygen (PiO_2_) of 100.9 ± 1.3 mmHg and hypobaric hypoxia conditions with a PiO_2_ of 106.0 ± 0.5 mmHg [45]. Understanding these distinctions is essential for accurately interpreting experimental outcomes and for designing interventions targeting specific hypoxic conditions.

Human studies conducted at high altitudes have linked hypobaric hypoxia to an exacerbation of gastrointestinal symptoms, including nausea, potential mucosal damage [46], and marked abdominal discomfort [47], all of which have been strongly associated with GER episodes. Abdominal discomfort appears to be influenced by factors such as age, sex, alcohol consumption, and smoking status [47]. Moreover, it is important to highlight that under hypobaric conditions, pharmacological strategies are used to mitigate or prevent certain pathological conditions in individuals at high altitude [48], such as acute mountain sickness (AMS) and high-altitude pulmonary hypertension (HAPH). The treatment for these diseases involves the use of drugs that reduce lower esophageal sphincter pressure, including calcium channel blocker agents [49], and, in the case of AMS, the use of aspirin and other nonsteroidal anti-inflammatory drugs, which increase the risk of developing GERD [19].

In addition, studies in patients with alterations in esophageal reflux have demonstrated a significant role for the stabilization of hypoxia-inducible factor subunits (HIF-1α and HIF-2α). In particular, HIF-2α can trigger the overexpression of proinflammatory mediators through reactive oxygen species (ROS) in this context. Furthermore, HIF-2α expression has been characterized as a prognostic biomarker of GER and the progression of Barrett’s esophagus to esophageal adenocarcinoma [50]. Prospective studies in 15 patients have shown that the stabilization of HIF-1α [16] and HIF-2α in patients with GER [51] under hypobaric hypoxia is associated with oxidative stress and inflammatory molecules [52], which may support the association between hypobaric hypoxia and GER through oxidative stress and proinflammatory proteins.

Finally, while UAO is clearly involved in the modulation of GER episodes, the direct role of hypoxia—particularly hypobaric hypoxia—in GER pathogenesis remains insufficiently understood. To the best of our knowledge, few studies have directly associated GER with hypobaric hypoxia. Therefore, further research is warranted to elucidate these complex interactions and to improve clinical management, especially in populations exposed to high-altitude conditions. Some recommendations can be useful for preventing or decreasing the secondary effects of SB; for example, the findings of Lile et al. [53] in 60 healthy adult volunteers suggest that natural mouth rinses containing propolis and green tea are effective at reducing dental plaque, with efficacy comparable to that of chlorhexidine. In the context of sleep bruxism, where increased masticatory activity and microtrauma can exacerbate oral and periodontal conditions, the use of such natural antimicrobial agents may help mitigate plaque accumulation and reduce the risk of secondary oral complications. This could be particularly relevant under hypoxic conditions, such as high-altitude exposure, where oxidative stress and autonomic dysregulation associated with bruxism may further compromise oral health.

## 5. Relationships Among Obstructive Sleep Breathing Disorders, Sleep Arousal and Sleep Bruxism

Various studies have associated obstructive sleep breathing disorders with sleep bruxism, but the exact relationship remains unclear [54,55,56,57]. Obstructive sleep breathing disorders (OSBD) are repetitive episodes of complete (apnea) or partial (hypopnea) UAO that occur during sleep. These events often result in reduced blood oxygen saturation and are usually terminated by sleep microarousal [58]. Approximately 50% of adult patients with certain types of OSBD have concomitant SB [59], but others report that the majority of apnea/hypopnea events are unrelated to SB events (84.5% of episodes) [60]. Nevertheless, the tonic activity of the masticatory muscles is evidenced in episodes of SB that occur immediately after or during episodes of apnea/hypopnea, facilitating the re-establishment of the airway and causing the termination of respiratory events [54,61]. Additionally, SB is linked to subtle forward movements of the lower jaw and tongue muscles, which expand the pharyngeal space and aid in the flow of air to the lungs [61].

Similarly, it has been postulated that the pathophysiology of OSA is intertwined with the pathogenesis of SB through the occurrence of microarousals during respiratory events [54,61]. For example, Yap et al. [60] reported that when apnea/hypopnea-SB events are related, SB events occur after apnea/hypopnea events and are predominant, suggesting a specific form of secondary SB triggered by sleep microarousals. This could be an important factor for individuals exposed to hypobaric hypoxia conditions. The collapsibility of the airway, whether partial or total, may be associated with desaturations in oxygen levels and subsequent arousals, which play a crucial role in initiating sleep [55]. The American Academy of Sleep Medicine defines arousals as an abrupt shift in electroencephalogram (EEG) frequency observed during polysomnography, including alpha, theta, and/or frequencies greater than 16 Hz, which lasts at least 3 s, with at least 10 s of stable sleep preceding the change. Rapid eye movement (REM) sleep is characterized by a concurrent increase in submental electromyography (EMG) lasting at least 1 s [62]. On the other hand, studies have shown that the sudden onset of RMMA during sleep occurs within a brief time when the brain is transitioning from sleep to an electroencephalographic aroused state [55,63]. Microarousals appear to be crucial in the sequence of events leading to the activation of the central pattern generator and jaw-closing muscles [63].

Moreover, Miki et al. [64] used standard nocturnal polysomnography in 14 healthy subjects (31.5 ± 5.7 years) to assess sleep quality and the presence of arousals and electromyography of the masseter muscle to identify episodes of rhythmic masticatory muscle activity (RMMA). They reported that all RMMA episodes coincided with sleep arousals and that movements such as lower limb movements, swallowing, and scratching were more frequent during episodes with RMMA. On the other hand, there is a significant association between sleep bruxism activity (RMMA/SB) and other body movements, particularly during arousals, suggesting a link between SB, arousal mechanisms, and physical movements. SB and OSA are associated with cardiovascular risks, potentially through mechanisms involving oxidative stress, inflammation, and autonomic nervous system activity [65,66,67]. In this way, through nocturnal polysomnography and venous blood samples, Fulek et al. [68] reported that a greater severity of SB is associated with increased oxidative stress markers, including elevated levels of advanced protein products (AOPPs) and thiobarbituric-acid-reacting substances (TBARSs), as well as a decreased total antioxidant capacity (TAC), with significant implications. These findings suggest that oxidative imbalance may contribute to the pathophysiology of SB. While some markers, such as 8-isoprostane, adiponectin, and leptin, did not show significant overall correlations, the oxidative stress profile—particularly increased oxidative damage and reduced antioxidant capacity—appears to play a role in the severity of SB. These results collectively suggest that oxidative stress markers could serve as indicators of SB severity and may be involved in its underlying mechanisms. However, these molecular signaling pathways and conditions have been well described for individuals exposed to hypobaric hypoxia. A study by Irarrázaval et al. [69] in mountaineers under conditions of acute hypobaric hypoxia, which simulated high-altitude conditions for 8 h, reported a significant increase in TBARS levels in the plasma of humans after 24 h of acute hypobaric hypoxia exposure. These findings are corroborated by preclinical studies under both intermittent (male Wistar rats exposed to 4600 m; 428 Torr) and chronic hypobaric hypoxia conditions (male Wistar rats exposed to 4600 m; 428 Torr) [70,71,72]. Furthermore, recent studies have shown a decrease in total TAC and an increase in oxidative biomarkers such as TBARSs and superoxide [73]. Therefore, on the basis of the above, we suggest that exposure to hypobaric hypoxia may contribute to both increased oxidative pathways and reduced antioxidant capacity, which are associated with SB.

Evidence linking sleep bruxism (SB) severity with increased oxidative stress markers, such as elevated levels of advanced oxidation protein products (AOPPs) and thiobarbituric-acid-reacting substances (TBARSs), along with reduced total antioxidant capacity (TAC), is clinically relevant [68,69,70,71,72,73]. Patients with SB who are exposed to hypobaric hypoxia—either due to high-altitude environments or intermittent hypoxic conditions—may be at heightened risk for oxidative stress-mediated complications, as hypoxia itself has been shown to exacerbate oxidative pathways and impair antioxidant defenses [69,70,71,72,73]. From a clinical perspective, these findings highlight the potential utility of monitoring oxidative stress biomarkers (e.g., TBARSs, TAC, and 8-isoprostane levels) in SB patients, particularly in those with hypoxia exposure, as indicators of both disease severity and systemic oxidative burden. Early detection of oxidative imbalance could inform the development of preventive or therapeutic strategies, such as antioxidant supplementation, lifestyle modifications, or interventions targeting hypoxia-related triggers, ultimately reducing the risk of long-term complications associated with SB and oxidative stress. Moreover, integrating polysomnography findings combined with biochemical profiling may allow clinicians to better stratify patients on the basis of oxidative stress status, facilitating more personalized management approaches for SB in hypoxic settings.

## 6. Microarousal-Induced Sleep Bruxism and Hypobaric Hypoxia Exposure

Although microarousals are defined as having sudden changes in brain wave frequency observed on polysomnography [74], current studies in male Sprague–Dawley rats under acute exposure to natural hypobaric hypoxia at 3270 m above sea level for 24 h (acute hypobaric hypoxia) have focused primarily on related aspects such as autonomic nervous system instability, cardiorespiratory function, and brain activation patterns [74]. Recent evidence in 12 young healthy women (mean age: 24.0 ± 4.2 years) exposed to hypobaric hypoxia (3500 m) for 4 days revealed reduced cardiac baroreflex sensitivity (cBRS), diminished vagal tone, and increased sympathetic activation—physiological conditions that favor the occurrence of microarousals during sleep [75]. Nevertheless, the direct relationship between microarousals and hypobaric hypoxia remains unexplored [76,77]. Future research should therefore employ polygraphic or polysomnographic studies at high altitude or in hypobaric chamber simulations, integrating EEG, cardiac baroreflex sensitivity, heart rate variability, respiratory parameters, and oxygen saturation to quantify the incidence, frequency, and severity of microarousals under hypoxic stress. Such studies would help clarify the neurophysiological pathways linking hypobaric hypoxia, autonomic dysregulation, and sleep fragmentation [75,76,77].

On the other hand, it has been proposed that the administration of melatonin improves sleep quality and the occurrence of such involuntary movements, which could be used as a preventive measure. In hypoxic contexts relevant to sleep bruxism, melatonin exhibits antioxidant and HIF-1α modulating properties and improves sleep continuity in individuals with COMISA/OSA, potentially reducing the number of microarousals that precede RMMA. Observational data in humans also suggest altered nocturnal melatonin in SB. While these findings in human studies support melatonin as a preventive adjunct targeting sleep stability, direct evidence that melatonin reduces SB/RMMA remains limited; hence, treatment of underlying sleep-disordered breathing should be prioritized, with melatonin considered an adjunct pending SB-specific trials [78,79].

On the other hand, recent studies of mountaineers (high-altitude exposure on Mount Blue Sky (4350 m), CO, USA) and humans, both adapted high-altitude dwellers (such as Andeans and Tibetans) and outsiders in the process of acclimatization at over 6000 m of altitude, highlight that respiratory support systems, including continuous positive airway pressure (CPAP), supplemental oxygen, and acetazolamide, effectively alleviate sleep-disordered breathing and improve sleep quality under high-altitude and hypobaric hypoxia conditions [80,81]. These interventions reduce the apnea–hypopnea index (AHI), increase oxygen saturation, and decrease the frequency of arousal, thereby enhancing sleep continuity and autonomic stability. Although no direct evidence currently links these interventions to reduced sleep bruxism (SB) or rhythmic masticatory muscle activity (RMMA), the pathophysiological overlap is compelling: SB episodes frequently follow microarousals, which are intensified by hypoxia-induced autonomic dysregulation. Thus, by mitigating hypoxia-related arousal and sleep fragmentation, respiratory support strategies may indirectly reduce SB occurrence. Future studies integrating polysomnography with masseter electromyography and autonomic markers should evaluate whether CPAP, oxygen supplementation, or acetazolamide confer protective effects against SB in hypoxic environments.

**Figure 1:**
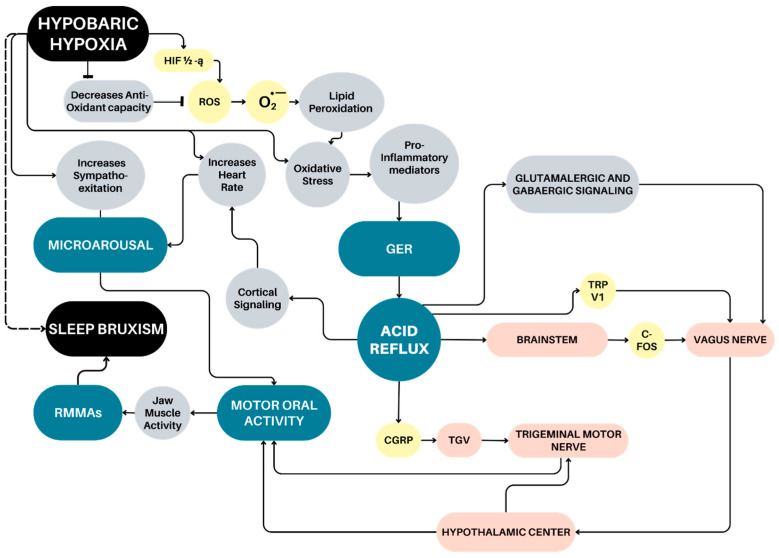
Hypothetical scheme of association with hypobaric hypoxia conditions and Sleep Bruxism (SB). HIF-1α (Hypoxia-Inducible Factor 1-alfa), ROS (Reactive Oxygen Species), O_2_ (molecular oxygen), GER (GastroEsophageal Reflux), TRPV1 (Transient Receptor Potential Vanilloid 1), c-FOS (cellular proto-oncogene Fos), CGRP (Calcitonin Gene-Related Peptide), TGV (Trigeminal Ganglion V) and RMMA (Rhythmic Masticatory Muscle Activity).

Finally, future research integrating sleep recordings (such as polysomnography) with these measurements under hypobaric hypoxia or high-altitude conditions (over 2500 m) is needed to confirm this association (Figure 1).

## 7. Conclusions

In conclusion, sleep bruxism (SB) is a multifactorial condition that can range from benign physiological behavior to a pathophysiological phenomenon influenced by gastroesophageal reflux (GER), autonomic instability, and hypobaric hypoxia exposure. Respiratory events such as airway obstructions and hypoxia act as key triggers for microarousals, which are subsequently associated with increased masticatory muscle activity. Additional factors—including psychological stress, genetic predisposition, sleep instability, and environmental influences such as high-altitude hypobaric hypoxia—further modulate the severity and frequency of SB episodes and related sleep disturbances. High-altitude environments, characterized by reduced atmospheric pressure and intermittent or chronic hypoxia, may exacerbate SB through sympathetic overactivation, sleep fragmentation, and an increased incidence of microarousal.

Oxidative stress appears to play a significant role in the pathophysiology of SB, with reactive oxygen species contributing to increased orofacial motor activity. Neurotransmitter systems, including brainstem reflex circuits and central pattern generators, mediate involuntary jaw muscle activity, whereas hypoxia and autonomic nervous system activation further modulate these processes. The potential protective role of SB in facilitating airway reopening, along with its interplay with central pattern generators and autonomic responses, underscores the multifaceted nature of this sleep phenomenon.

Molecular interactions between GER and neural pathways highlight the complex biochemical environment contributing to SB episodes. GER, which may be aggravated under hypoxic conditions, can trigger a brainstem-mediated protective sequence known as the “triple event model”, consisting of acid reflux, cortical arousal, and rhythmic masticatory muscle activity. Hypoxia-inducible factors (HIFs), nociceptive receptors, and neuropeptides contribute to neuroplastic changes and increased orofacial motor responses. While further studies are warranted, the current evidence supports a plausible link between hypobaric hypoxia and increased SB activity, emphasizing the need for integrated diagnostic approaches and targeted preventive strategies, particularly for individuals exposed to high-altitude environments.

### Clinical Implications

Understanding the multifactorial contributors to SB can guide individualized patient management. Assessing oxidative stress and autonomic responses may help identify patients at greater risk of severe SB and related systemic complications. Differentiating exposure to normobaric versus hypobaric hypoxia is essential in high-altitude settings. Integrated management strategies should address GER control, sleep stabilization, and a reduction in sympathetic overactivation. Polysomnography remains the gold standard for SB evaluation, but studies directly assessing SB at high altitude are limited, highlighting the need for further research to translate mechanistic insights into clinical practice.

## Data Availability

Not applicable.

## Abbreviations

The following abbreviations are used in this manuscript:

SB	Sleep bruxism
OSA	Obstructive Sleep Apnea
CIHH	Chronic Intermittent Hypobaric Hypoxia
PaO_2_	Partial Pressure of Oxygen
TG	Tooth Grinding
GER	Gastroesophageal Reflux
LES	Lower Esophageal Sphincter
RMMA	Rhythmic Masticatory Muscle Activity
TRPV1	Transient Receptor Potential Vanilloid 1
NTS	Nucleus Tractus Solitarius
MotN	The trigeminal motor Nucleus
TGV	Trigeminal Nerve
c-FOS	Cellular-Fos
CGPR	Calcitonin Gene-Related Peptide
HIF-1α	Hypoxia-inducible factors
NA	Noradrenaline
UAO	Upper Airway Obstruction
ROS	Reactive Oxygen Species
OSBD	Obstructive Sleep Breathing Disorders
OSA	Obstructive Sleep Apnea Syndrome
EEG	Electroencephalogram
REM	Rapid Eye Movement
EMG	Electromyography
CAP	Cyclic Alternating Pattern
AOPP	Advanced Protein Products
TBARS	Thiobarbituric Acid-Reacting Substances
TAC	Total Antioxidant Capacity
cBRS	Reduced Cardiac Baroreflex Sensitivity
